# MGL-YOLO: A Lightweight Barcode Target Detection Algorithm

**DOI:** 10.3390/s24237590

**Published:** 2024-11-27

**Authors:** Yuanhao Qu, Fengshou Zhang

**Affiliations:** School of Mechatronics Engineering, Henan University of Science and Technology, Luoyang 471003, China; yuanhaoqu@stu.haust.edu.cn

**Keywords:** one-dimensional barcode recognition, target detection, lightweight network, feature extraction, deep learning

## Abstract

Due to the critical importance of one-dimensional barcode detection in logistics, retail, and manufacturing, which has become a key issue affecting operational efficiency, researchers have shown increasing interest in this area. However, deploying deep convolutional neural networks on embedded and some edge devices is very challenging due to limited storage space and computational resources. To address this issue, this paper proposes MGL-YOLO, a lightweight one-dimensional barcode detection network based on an improved YOLOv8, which aims to achieve a high detection accuracy at low computational cost. First, a new multi-scale group convolution (MSGConv) is designed and integrated into the C2f module to construct the MSG-C2f feature extraction module. By replacing the C2f module in the P5 layer of the backbone network, the ability to extract multi-scale feature information is enhanced. Secondly, a feature extraction module, Group RepConv Cross Stage Partial Efficient Long-Range Attention Network (GRCE), is designed to optimize the feature extraction capability of the C2f modules in the neck section, offering significant advantages in multi-scale characteristics and complexity adjustment. Finally, a Lightweight Shared Multi-Scale Detection Head (LSMD) is proposed, which improves the model’s detection accuracy and adaptability while reducing the model’s parameter size and computational complexity. Experimental results show that the proposed algorithm increases MAP50 and MAP50.95 by 2.57% and 2.31%, respectively, compared to YOLOv8, while reducing parameter size and computational cost by 36.21% and 34.15%, respectively. Moreover, it also demonstrates advantages in average precision compared to other object detection networks, proving the effectiveness of MGL-YOLO for one-dimensional barcode detection in complex backgrounds.

## 1. Introduction

One-dimensional barcodes, which encode numbers and characters in bar patterns, are widely used across various fields [1]. In recent years, with the development of the modern economy, the applications and demand for one-dimensional barcodes have increased significantly, leading to stricter requirements for their recognition. During the processing, transportation, and storage of products or goods with one-dimensional barcodes, issues such as dirt, damage, occlusion, and image blurring can adversely affect the recognition performance, accuracy, and efficiency of barcodes. Additionally, the large size of existing high-precision detection models can impact deployment costs. Therefore, proposing a new lightweight network that can accurately and efficiently recognize one-dimensional barcodes in complex scenarios has become essential.

In the early stages of one-dimensional barcode recognition, a scanner and a decoder were typically used [2]. The scanner emitted light and received reflected light, while the decoder was capable of determining the reflected signal bars. However, this method was inefficient and prone to missed and erroneous detections. To address the numerous challenges faced in one-dimensional barcode recognition, using deep learning as a detection method has become a necessary choice.

In recent years, numerous scholars have conducted extensive research on one-dimensional barcode recognition and have achieved notable results. Daniel Kold Hansen et al. [3] applied YOLO to one-dimensional barcode detection, enabling rotation detection with high accuracy. Savreet Kaur et al. [4] proposed a barcode localization method based on basic morphology. Andrey Zharkov et al. [5] developed a new fast and robust deep learning detector based on semantic segmentation, capable of detecting barcodes of any type simultaneously in both document scans and real-world conditions using a single model. Jun Jia et al. [6] proposed a new method for efficient barcode detection in real and complex environments using a convolutional neural network (CNN) model, which improves decoding rates by eliminating distortions. Qijie Zhao et al. [7] introduced BarcodeNet, a segmentation network based on a dual-pyramid structure, which maintains high precision in challenging conditions such as small-scale targets, partial occlusion, shape deformation, and significant lighting changes. Despite the high accuracy achieved by the deep learning models mentioned above in barcode recognition, their large parameter sizes and high computational resource consumption highlight the urgent need for the design of a lightweight neural network to meet the requirements of resource-constrained devices.

Many scholars have also provided valuable research contributions to lightweight networks. Dehua Zhang et al. [8] designed an efficient lightweight convolutional neural network (CNN) model for surface defect detection in industrial products, achieving a detection accuracy comparable to state-of-the-art models while reducing parameters and computational demands. D.N. Kiran Pandiri et al. [9] proposed a novel lightweight CNN for soil type prediction, demonstrating high accuracy in soil classification. Jia Liang et al. [10] developed a lightweight segmentation model based on PCSNet, effectively overcoming the performance limitations caused by sampling operations that lead to feature extraction and boundary loss, thereby enhancing PCSNet’s feature extraction capabilities. Dehua Zhang et al. [11] also introduced a novel lightweight deep CNN algorithm for SDD, which achieves higher precision and speed with fewer computational parameters, and exhibits excellent generalization capabilities. Hua Shen et al. [12] proposed L-Net, a lightweight convolutional neural network specifically designed for low-computation devices, contributing theoretically to the lightweight deep learning (DL) field. Yang Chen et al. [13] introduced DFCANet (Double Fusion block with Coordinate Attention Network), a lightweight corn disease recognition model that improves the efficiency of detecting corn leaf diseases in real-world images. Fu-Jun Du et al. [14] proposed BV-YOLOv5S, an enhanced lightweight object detection algorithm based on the YOLO framework, which performs well in road surface defect detection, meeting the high-level requirements for real-time performance and flexibility in road safety inspection projects. Huafeng Wu et al. [15] developed a lightweight object recognition technique based on an improved YOLOv4-tiny algorithm, enabling the precise and efficient detection of ship fires while addressing the distinct characteristics of ship fires and the marine environment. Mengjia Yan et al. [16] introduced VarGFaceNet, an efficient variable group convolution network that enhances the decision-making and generalization capabilities of lightweight face recognition networks. Peng Xiao et al. [17] proposed a new framework for fast tumor identification (TFI) in mobile computer-aided devices, comprising a weight accumulation method and a lightweight fast neural network (FastNet). Due to the complex and diverse application environments of barcodes, and the large-scale variations of different barcodes, existing lightweight networks are not well-suited for barcode recognition in complex backgrounds.

In complex environments such as those with dirt, damage, and occlusion, achieving ideal results remains challenging. Additionally, issues related to excessive parameter and computational complexity persist. This paper improves upon YOLOv8, ensuring that the model remains lightweight while enhancing the accuracy of one-dimensional barcode detection and providing advantages in complex environments. The improvements mainly include the following three aspects:

1. To address the challenges of large-scale transformations and complex barcode images that lead to poor detection performance, this paper introduces MSGConv convolution to improve the C2f module, resulting in the MSG-C2f module. The MSGConv module uses convolution layers with different kernel sizes to capture features at various scales and employs group convolution to reduce computational costs. Features are integrated through 1 × 1 convolution layers, maintaining high computational efficiency. This modification enhances the feature extraction capability of the backbone network’s P5 layer C2f.

2. To tackle the issues of limited feature extraction depth, low flexibility, and poor performance in handling complex backgrounds and details in the C2f feature extraction module of the neck structure, a new feature extraction module, GRCE, is proposed. The GRCE module allows for the flexible adjustment of the model’s complexity and computational cost, effectively reducing the computational load and parameter size while extracting and fusing multi-scale features. Replacing the neck structure of the original C2f modules with that of GRCE modules improves the neck’s feature fusion capability, making it more suitable for complex scenarios.

3. To address the issues of large parameter size, high complexity, and inability to capture multi-scale features in the original detection head, a lightweight object detection head, LSMD, was designed. The LSMD detection head uses group normalization convolution (Conv_GN) and multi-scale group convolution (MSGConv) to enhance detection stability and accuracy. Additionally, the LSMD detection head employs shared convolution, allowing each detection layer to share some convolution operations, resulting in a reduction in parameter size and computational complexity.

## 2. Methods

### 2.1. YOLOv8 Architecture Network

YOLOv8 (You Only Look Once version 8) is an end-to-end single-stage object detection algorithm that performs both object localization and classification in a single forward pass [18]. YOLOv8 utilizes Darknet-53 as its backbone network, which extracts image features through multiple convolutional layers and residual blocks, and incorporates a Feature Pyramid Network (FPN) to merge features from different levels, thereby improving the detection accuracy. During object prediction, YOLOv8 divides the image into multiple grids and predicts the object classes and bounding boxes within each grid. To enhance the accuracy of the detection results, YOLOv8 employs post-processing methods such as Non-Maximum Suppression (NMS) and confidence thresholds to eliminate overlapping bounding boxes, retaining only high-confidence predictions, and ultimately provides bounding boxes with information on object location, class, and confidence.

**Figure 1:**
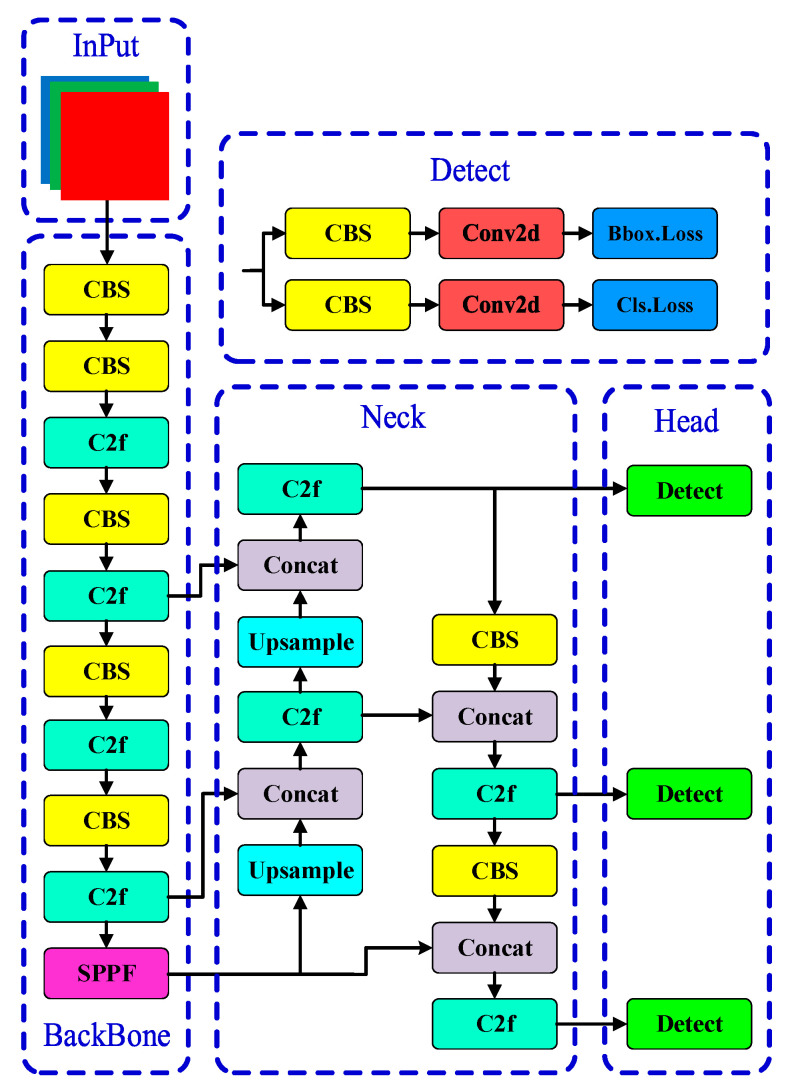
YOLOv8 (You Only Look Once version 8) model structure. In the backbone, multiple convolutional layers and residual blocks are employed for effective feature extraction. The neck part incorporates the C2f module to enhance feature fusion, while the Head consists of separate detection branches for object classification and localization, facilitating efficient detection output.

The YOLOv8 network architecture is typically divided into three functional parts: the Backbone for feature extraction, the neck for feature fusion at different scales, and the Head for object localization and classification [19]. As shown in Figure 1, the backbone section replaces the C3 module with the C2f module, reducing the network’s parameter size while enhancing the feature extraction capability. The SPPF module is also incorporated, and models are fine-tuned at different scales, significantly improving performance. In the neck section, the C2f module replaces the C3 module, and removed the 1 × 1 convolutional layer before upsampling, which achieves model lightweighting while enhancing the fusion of multi-scale feature layers. In the Head section, YOLOv8 employs a Decoupled-Head structure, separating the classification and detection heads. Additionally, it replaces Anchor-Based methods with Anchor-Free methods, reducing computation time and power, and decreasing the probability of missed and duplicate detections.

### 2.2. Improved YOLOv8 Barcode Detection Model

**Figure 2:**
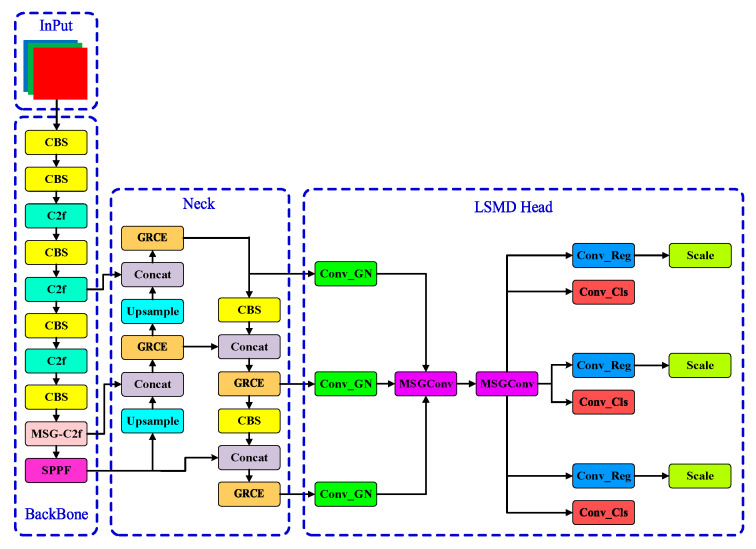
Structure of MGL-YOLO (MSG-C2f GRCE LSMD-YOLO) mode. It integrates the MSG-C2f module in the backbone for improved multi-scale feature extraction. The GRCE module in the neck enhances feature fusion capabilities, and the LSMD detection head optimizes parameter efficiency while maintaining high detection accuracy, particularly for barcodes in complex environments.

To reduce the model’s parameter size and computational load while enhancing the detection accuracy, this paper proposes an improved YOLOv8-based model called MGL-YOLO. The model architecture is shown in Figure 2. MGL-YOLO is a lightweight one-dimensional barcode detection network, designed to optimize the balance between model size and detection accuracy, improving the network’s capability to detect barcodes in complex scenarios. First, the MSGConv convolution is used to replace the C2f module in the backbone network’s P5 layer with the MSG-C2f module. By employing group convolutions with different scales, this approach reduces the number of parameters and lowers the rate of missed detections caused by barcode scale transformations. Second, the proposed GRCE module is used to replace the C2f modules in the original network’s neck structure, enhancing the network’s flexibility and feature extraction capability, thus improving the detection accuracy. Finally, a lightweight detection head, LSMD, is introduced. LSMD utilizes shared convolutions to further reduce the parameter size and computational complexity. The inclusion of MSGConv convolution allows the model to better capture multi-scale features, thereby enhancing the detection accuracy.

#### 2.2.1. MSG-C2f Feature Extraction Module

**Figure 3:**
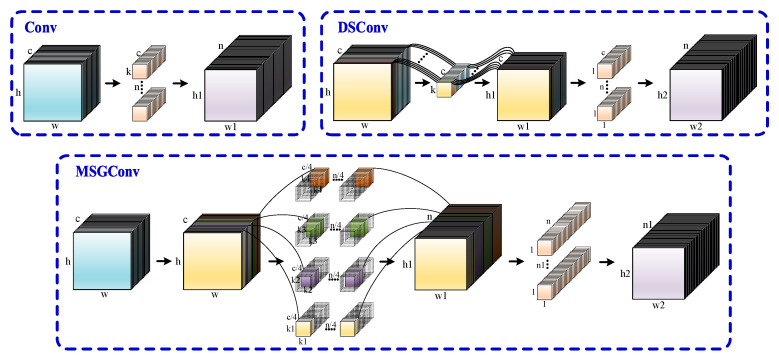
Comparison of Different Convolutional Implementation Principles. This image compares the principles of standard convolution, DSConv (depthwise separable convolution), and the proposed MSGConv (multi-scale group convolution). MSGConv divides input channels into groups, applying different kernel sizes to extract features at various scales, which not only reduces parameter count but also improves detection accuracy.

(1) MSG Convolution;

Unlike traditional standard convolution (Conv) and popular depthwise separable convolution (DSConv), this paper introduces a multi-scale group convolution called MSGConv, as shown in Figure 3. MSGConv employs the concept of grouping by dividing the input channels into four groups, each using convolution kernels of different sizes. Compared to standard convolution, this approach not only reduces the number of parameters but also extracts features at multiple scales, thereby improving the detection accuracy. Unlike traditional standard convolution (Conv) and popular depthwise separable convolution (DSConv), this paper introduces a multi-scale group convolution called MSGConv, as shown in Figure 3. MSGConv employs the concept of grouping by dividing the input channels into four groups, each using convolution kernels of different sizes. Compared to standard convolution, this approach not only reduces the number of parameters but also extracts features at multiple scales, thereby improving the detection accuracy.

As shown in Figure 3, the dimensions of the image change after convolution. The formula for calculating the size after convolution is as follows:

(1) $$\left\{\begin{array}{l} w1=\frac{w-k+2p}{s}+1\\ h1=\frac{h-k+2p}{s}+1 \end{array}\right.$$

where *w* and *w*1 represent the width of the image before and after convolution, respectively, *h* and *h*1 represent the height of the image before and after convolution, respectively, *k* denotes the size of the convolutional kernel, *p* is the padding size, and *s* is the stride. The formula for calculating the number of parameters in standard convolution is:

(2) params(Conv)=(k×k×c+1)×n

where params represents the number of parameters in the convolution, *k* is the size of the convolution kernel, *c* is the number of channels in the convolution, and *n* is the number of kernels. The formula for calculating the computational cost of standard convolution is as follows:

(3) FLOPs(Conv)=(k×k×c+1)×n×w1×h1

where *w*1 and *h*1 represent the width and height of the image after convolution. The formula for calculating the number of parameters in the *MSGConv* convolution is as follows:

(4) $$params(MSGConv)=\sum_{i=1}^{4}\left(ki\times ki\times \frac{c}{4}+1\right)\times \frac{n}{4}$$

where *ki* represents the i-th convolution kernel. Since *MSGConv* divides the input channels into four groups, *MSGConv* uses four convolution kernels of different sizes. *c*/4 and *n*/4 represent the number of channels and the number of kernels for each convolution. The formula for the computational complexity of *MSGConv* is as follows:

(5) $$FLOPs(MSGConv)=\sum_{i=1}^{4}\left(ki^{2}\times \frac{c}{4}+1\right)\times \frac{n}{4}\times w1\times h1$$

From the above formula, it can be observed that when the convolution kernel size *ki* of *MSGConv* is the same as the convolution kernel size *k* of a standard convolution (Conv), the parameter count and computational cost of *MSGConv* are approximately only 1/4 of that of the standard convolution. In this work, *MSGConv* uses four groups with convolution kernel sizes of 1 × 1, 3 × 3, 5 × 5, and 7 × 7. Compared to a 3 × 3 standard convolution, the parameter count and computational cost are reduced by approximately 41.7%.

**Figure 4:**
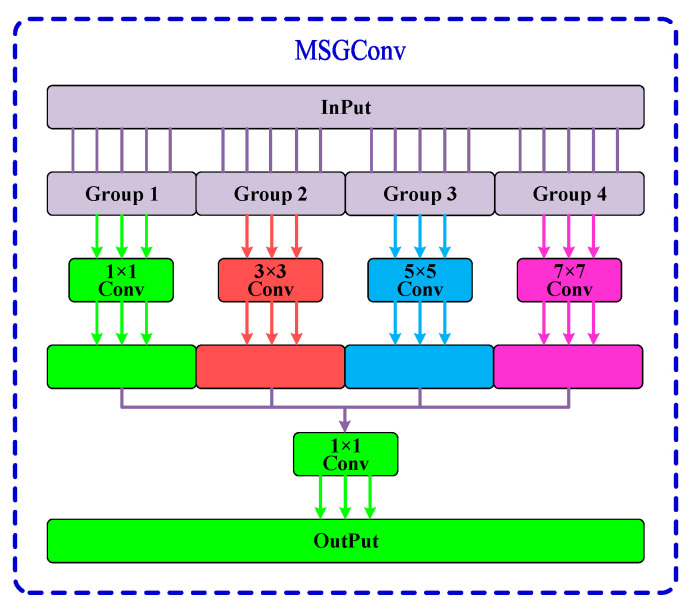
MSGConv structure. This image depicts the structure of MSGConv, highlighting its ability to utilize multiple convolutional kernel sizes to capture multi-scale features. By grouping input channels and applying different convolution operations, MSGConv effectively enhances the model’s ability to recognize barcodes across varying sizes and complexities.

After grouping, different convolution kernel sizes are applied to each group to capture multi-scale information. The structure of *MSGConv* is shown in Figure 4.

MSGConv can adjust the receptive field range and multi-scale information by modifying the convolution kernel size, enabling more accurate barcode recognition. Finally, a 1 × 1 convolution is used to fuse information across groups, keeping the number of channels constant while achieving lightweight and efficient feature aggregation.

**Figure 5:**
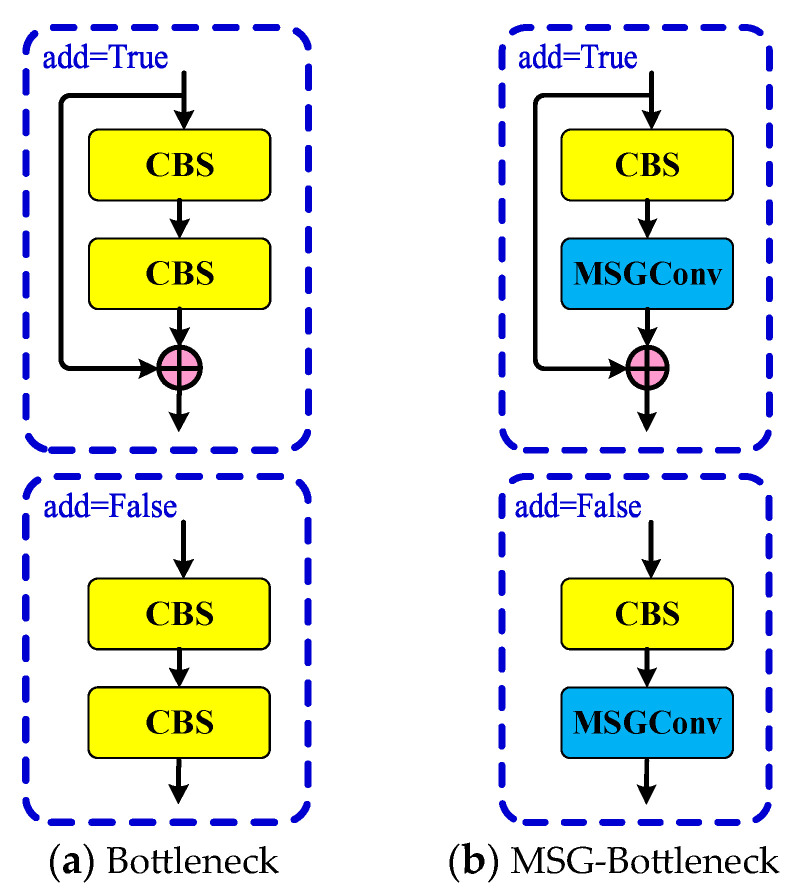
Comparison of Bottleneck before and after improvement. The enhancement involves replacing standard convolutions with MSGConv, which leads to a reduction in parameters and an increase in multi-scale feature extraction capability, thereby improving barcode detection performance in challenging environments.

(2) MSG-C2f Module;

Based on the lightweight characteristics of MSGConv and its strong multi-scale feature extraction capability, improvements have been made to the Bottleneck module within the C2f module. The original Bottleneck module is shown in Figure 5a. By replacing the second CBS module in the Bottleneck with an MSGConv convolution, a structure called MSG-Bottleneck is created, as shown in Figure 5b. A comparison of the Bottleneck structure before and after the improvement is depicted in Figure 5.

**Figure 6:**
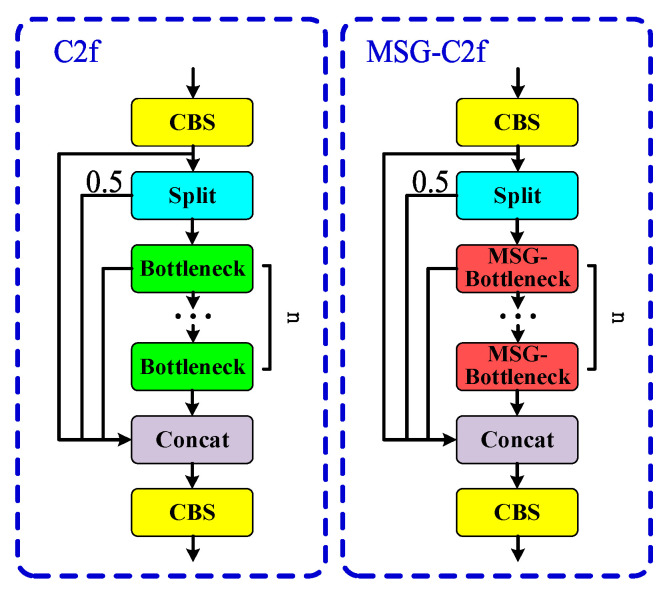
Comparison of C2f before and after improvement. The modified C2f module benefits from enhanced feature extraction and reduced computational complexity, allowing for more efficient processing of barcodes in complex scenarios.

Replacing the original Bottleneck in the C2f module with MSG-Bottleneck can effectively reduce the number of parameters and enhance the backbone network’s ability to extract multi-scale features, as well as improve the barcode detection performance in complex environments. The C2f module before and after the improvement is shown in Figure 6.

The task of barcode detection often involves various complex scenarios, such as significant differences in barcode size, shape, and rotation angle. Barcodes may also be partially occluded, damaged, or blurred, with complex backgrounds that may confuse the barcode with background textures. In these situations, traditional convolutions struggle to capture information at different scales and levels of detail due to their fixed receptive fields and kernel sizes. MSGConv addresses this issue by utilizing multi-scale group convolution with kernels of different sizes (such as 1 × 1, 3 × 3, 5 × 5, and 7 × 7) to capture features at various scales, ensuring accurate barcode detection even in complex environments. Additionally, MSGConv not only introduces multi-scale feature extraction but also employs group convolution to reduce the computational complexity. Unlike standard convolutions, group convolution divides the input feature map into multiple groups, with each group being processed independently. This approach significantly reduces the number of parameters while improving the computational efficiency, which is particularly beneficial for processing high-resolution images or operating on edge devices with limited computing resources.

**Figure 7:**
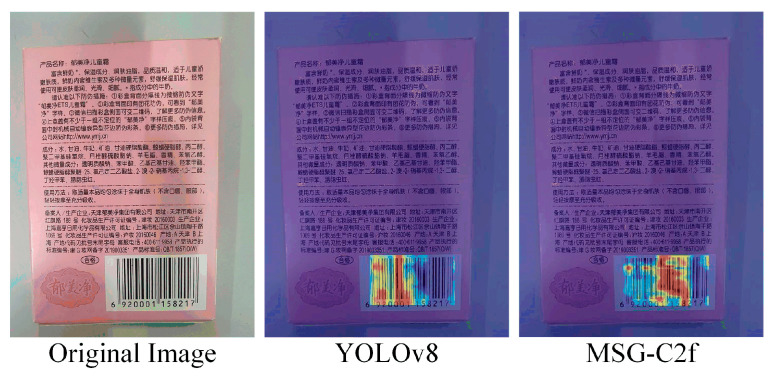
Comparison of heatmaps before and after improvement. Heatmap comparisons were made between YOLOv8 and the improved MSG-C2f. It can be observed that the improved model demonstrates clear advantages over YOLOv8.

To visually demonstrate the effects before and after the improvements, this paper includes a heatmap comparison, as shown in Figure 7.

The heatmap clearly shows that the improved MSG-C2f detects barcodes more accurately than YOLOv8, indicating that MSG-C2f enhances the feature extraction capability of the model, thereby validating the effectiveness of the algorithm.

#### 2.2.2. GRCE Feature Extraction Module

**Figure 8:**
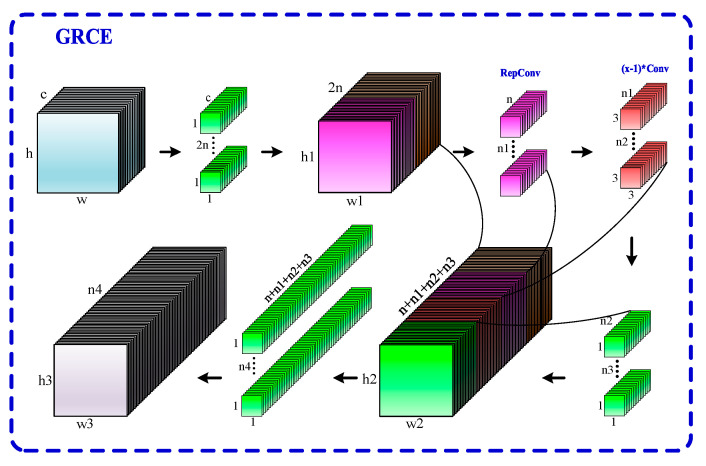
Implementation principle of the GRCE (Group RepConv Cross Stage Partial Efficient Long–Range Attention Network) module. It first applies a 1 × 1 convolution to double the number of hidden channels, then splits the feature map for deeper processing. This design enhances feature diversity and enables flexible adjustments to the model’s complexity, leading to better barcode detection.

In the YOLOv8 model, unlike the backbone network, which is responsible for feature extraction, the neck structure typically plays a role in feature fusion. The C2f module, which consists of only two convolutional layers and *n* Bottleneck modules, exhibits good feature extraction capability. However, its feature fusion capability and diversity are relatively poor, particularly in complex backgrounds, leading to a lower detection accuracy. Additionally, the C2f module uses fixed kernel sizes, lacking multi-scale convolution operations, which may result in missing useful information. Therefore, this paper proposes a more efficient, flexible, and lightweight GRCE module to replace the original C2f module in the neck structure. The principle of the GRCE module is illustrated in Figure 8.

**Figure 9:**
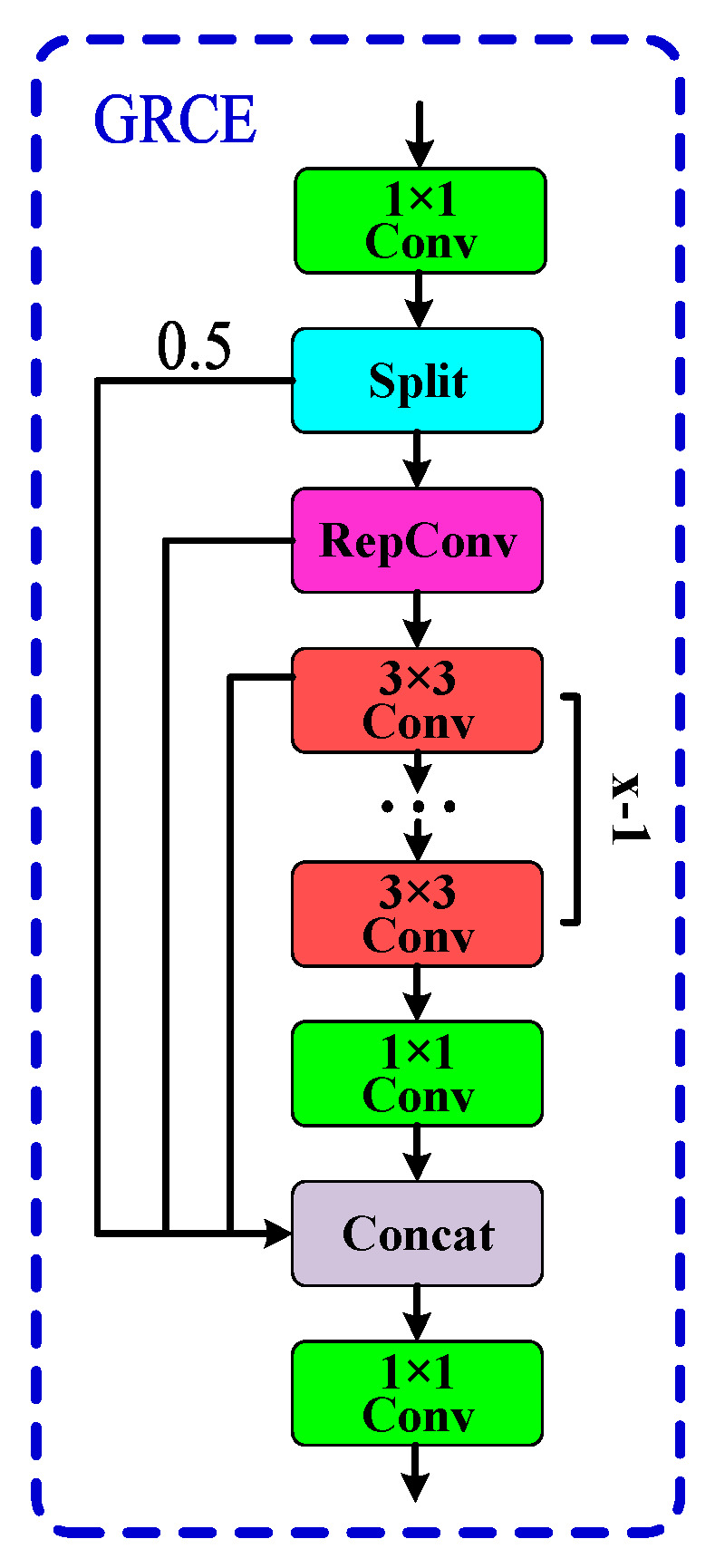
GRCE Module Structure. The GRCE module begins with a 1 × 1 convolution to double the number of channels, followed by a split to process features through both direct and deeper pathways. This design enhances the model’s flexibility and enables it to capture richer feature information, which is essential for effective barcode detection in complex backgrounds.

As shown in Figure 8, the GRCE module first applies a 1 × 1 convolution operation to the input image, which transforms the input channels into twice the number of hidden layer channels. The resulting feature map is then split into two parts. One part is passed directly to the subsequent layers without modification, while the other part undergoes a sequence of RepConv convolutions and x−1 3 × 3 convolutions to extract deeper feature information. After this, a 1 × 1 convolution is applied for integration and channel conversion. By merging the information from each convolution operation during the process, the GRCE module retains the original information while obtaining features at different depths. Finally, another 1 × 1 convolution is used for feature fusion, resulting in a more comprehensive and richer feature map. The specific structure of the GRCE module is illustrated in Figure 9.

A common challenge in barcode detection tasks is the presence of complex, noisy backgrounds, especially in application scenarios like warehousing and logistics, where barcodes can easily be confused with other objects in the background, such as text, lines, or shapes. The GRCE module (Group RepConv Cross Stage Partial Efficient Long-Range Attention Network) enhances feature extraction diversity and long-range dependency awareness, allowing the model to better differentiate barcodes from the background, thereby reducing the likelihood of false detections. Barcodes often face partial occlusion or damage during detection. The GRCE module addresses this by leveraging a cross-stage partial connection mechanism, which preserves some of the original feature information and combines it with features processed through deep convolution. This improves the model’s ability to handle occluded or damaged barcodes. In environments such as logistics or supermarkets, barcodes may become blurred due to issues with capturing devices (e.g., camera shake or poor lighting). The GRCE module, through its efficient feature processing using the RepConv structure during inference, enables the model to strike a balance between inference speed and accuracy, allowing it to quickly and accurately detect barcodes even in blurred conditions.

**Figure 10:**
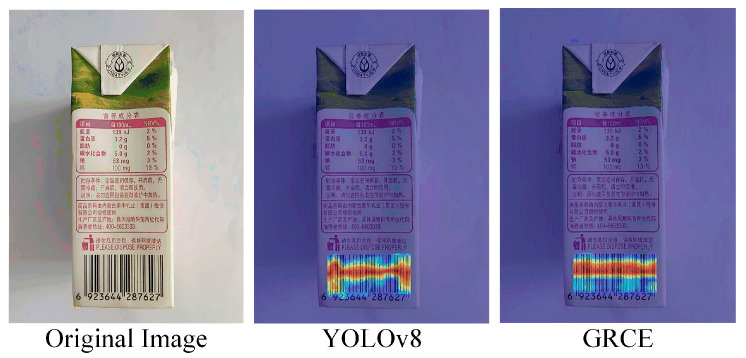
Comparison of the GRCE module before and after improvements. After comparing the heatmaps before and after adding the GRCE module, it was evident that the improved network exhibits a more stable detection performance that aligns better with actual detection results.

To visually assess the detection performance of the GRCE module, heatmap comparisons were made between the original and improved YOLO models, as shown in Figure 10.

**Figure 11:**
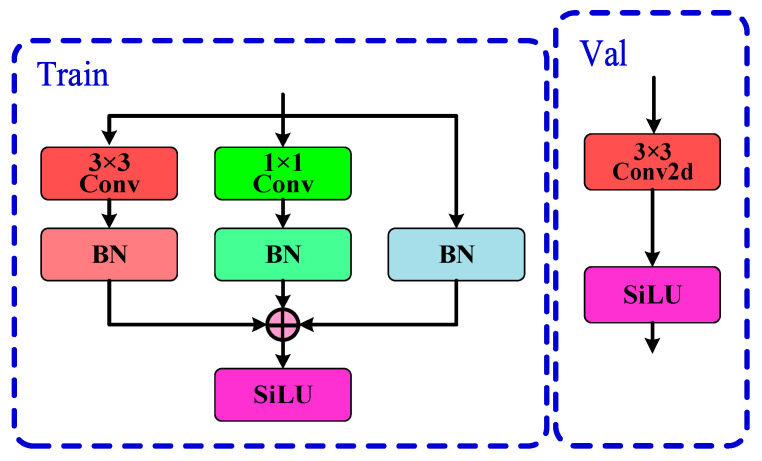
RepConv module structure, which utilizes a multi-branch architecture during training to enhance feature extraction. During inference, it transforms into a single-branch structure, optimizing computational efficiency while maintaining accuracy. This innovative approach allows for effective learning of object characteristics, particularly useful for detecting barcodes in various conditions.

The GRCE module can adjust the Split value as needed to modify the model’s detection accuracy and computational load, offering high flexibility. In this study, the default Split value is set to 0.5. The introduction of RepConv convolutions enhances feature diversity and richness, improving the model’s expressiveness and computational efficiency [20]. As shown in Figure 11, RepConv uses a multi-branch structure during training to boost the detection accuracy. During inference, it is equivalently transformed into a single-branch structure through techniques such as structure reparameterization, thus improving inference speed while maintaining accuracy.

In network models, there are typically many convolution and batch normalization operations. By merging convolution layers and normalization layers, the number of layers in the network model can be reduced, thereby improving operational efficiency. Let the input feature map be denoted as *x* ∈ *R^N×C×H×W^*, where N represents the batch size, *C* denotes the number of channels, and *H* and *W* represent the height and width of the feature map, respectively. Convolution layers usually include the bias term, Bias, and the convolution operation is expressed as:

(6) Conv(x)=W(x)+b

where *W*( ) represents the nonlinear convolution operation, and the Bias term of the convolution layer is denoted as *b*. The calculation formula for the batch normalization layer (*BN*) can be expressed as:

(7) $$BN(x)=\gamma \times \frac{x-\mu }{\sigma }+\beta$$

where *μ* represents the mean of the samples, *σ* represents the variance of the samples, *γ* denotes the learned scaling factor, and *β* denotes the bias. The result of the convolution layer after batch normalization (*BN*) can be expressed as:

(8) $$BN(Conv(x))=\gamma \times \frac{W(x)+b-\mu }{\sigma }+\beta$$

Equation (8) can be transformed into:

(9) $$BN(Conv(x))=\frac{\gamma \times W(x)}{\sigma }+(\frac{\gamma \times (b-\mu )}{\sigma }+\beta )$$

Based on Equation (9), let:

(10) $$\left\{\begin{array}{l} W_{f}=\frac{\gamma \times W()}{\sigma }\\ b_{f}=(\frac{\gamma \times (b-\mu )}{\sigma }+\beta ) \end{array}\right.$$

where *Wf* and bf represent the convolution kernel weight parameters and bias after batch normalization (*BN*), respectively. The result of merging the convolution layer and batch normalization can be expressed as:

(11) $$BN(Conv(x))=W_{f}(x)+b_{f}$$

At this point, the parameter fusion of the convolution layer and batch normalization (*BN*) layer has been achieved.

**Figure 12:**
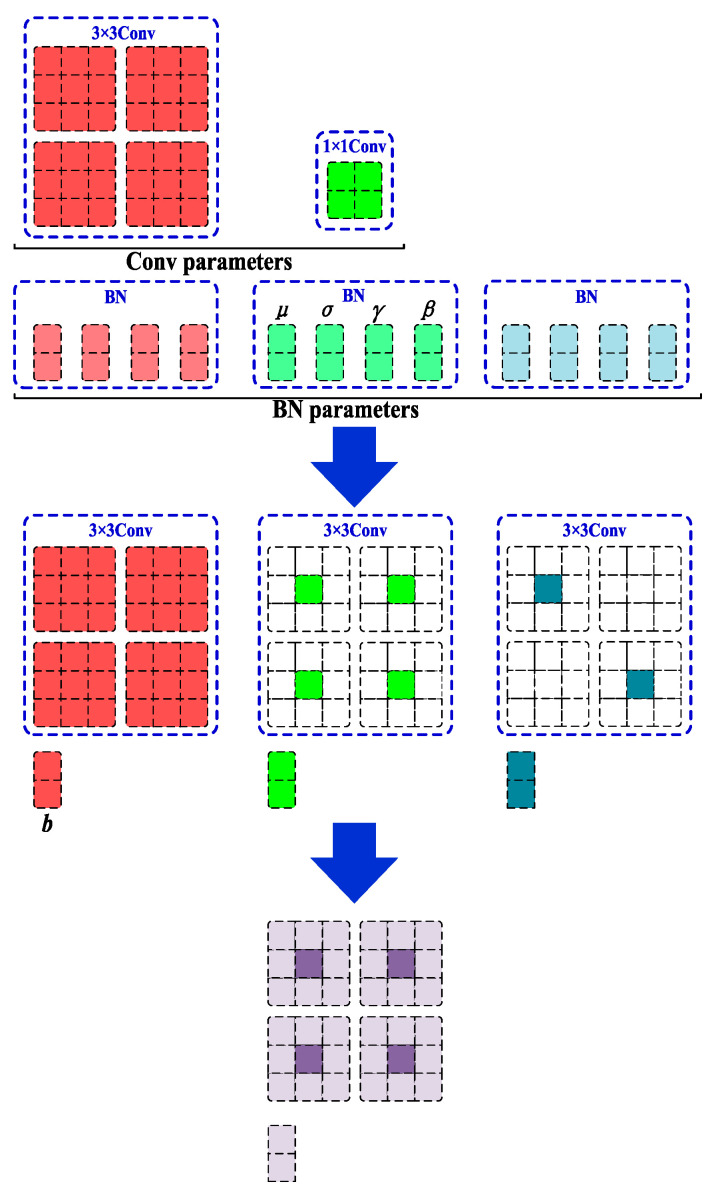
RepConv Parameter Structure, which merges convolutional and batch normalization layers to improve operational efficiency. By reducing the number of layers, the RepConv module allows for faster inference while maintaining the accuracy of the detection model.

From Figure 12, it can be seen that a 1 × 1 convolution kernel can be expanded into a 3 × 3 convolution kernel by surrounding it with 0 padding. Additionally, a residual structure can be used to construct four convolution kernels, where only two of the kernels have a central value of 1, while the rest have all values set to 0. According to the additivity principle of convolution, multiple 3 × 3 convolution kernels can be equivalent to a single 3 × 3 convolution kernel, which effectively reduces the parameter count of the network structure.

#### 2.2.3. LSMD Detection Head

The detection head module is a critical component in object detection models, responsible for converting feature maps generated by the feature extraction network into object category predictions and bounding box locations. It directly impacts the prediction accuracy, efficiency, and adaptability of the object detection process. In YOLOv8, while the original detection head performs well in many aspects, it has some shortcomings in handling small objects and also has a large parameter count, accounting for approximately one-quarter of the entire model’s parameters. To improve computational efficiency and better accommodate objects of various sizes, this paper proposes a lightweight detection head, LSMD, aimed at making the model suitable for memory-constrained edge devices.

**Figure 13:**
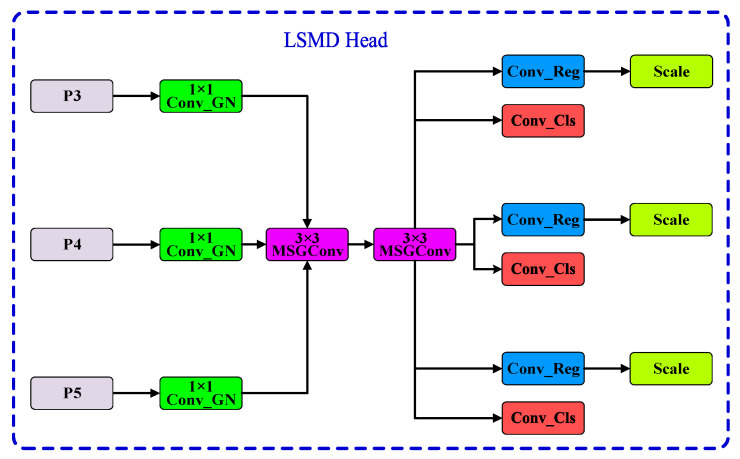
LSMD (Lightweight Shared Multi-Scale Detection) structure, which employs shared convolution operations among different detection branches. This design reduces the overall parameter count while enabling effective multi-scale feature extraction, thus enhancing the model’s adaptability for barcode detection tasks.

Compared to traditional detection heads, the main highlight of the LSMD detection head is its use of parameter sharing. Unlike traditional detection heads that perform separate computations for each detection branch, the LSMD detection head shares data among modules such as MSGConv, Conv_Reg, and Conv_Cls. In other words, multiple detection branches perform computations only once, significantly reducing both parameter count and computational complexity. To meet the detection needs of features at different scales, a Scale module is used to perform feature scaling for each detection branch, enabling the detection of one-dimensional codes at various scales. The LSMD structure is shown in Figure 13.

From Figure 13, it can be seen that the LSMD structure first applies a combination of convolution layers and group normalization layers, Conv_GN (Conv_Group Normalization), to adjust the number of channels and perform feature fusion. The introduction of group normalization layers enhances the stability of the model [21]. Then, the MSGConv module is used for multi-scale feature extraction and fusion. The fused multi-scale feature maps are processed separately by Conv_Reg and Conv_Cls for bounding box regression and class prediction, respectively. Finally, the Scale module performs feature scaling to achieve the detection of features at different scales.

**Figure 14:**
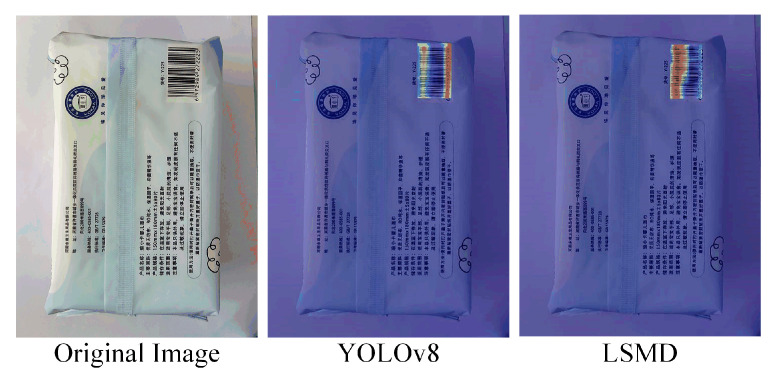
Heatmap comparison before and after the LSMD improvement. The improved heatmap demonstrates greater accuracy, as seen in the figure.

Heatmap comparison before and after adding the LSMD detection head, as shown in Figure 14.

Compared to the baseline model, the improved model exhibits a more accurate detection performance, which also validates the significant advantages of the LSMD in barcode detection and proves the effectiveness of the improvements.

A core challenge in barcode detection tasks is that barcodes are typically small in size, and traditional detection heads often struggle to accurately detect small objects. The LSMD detection head (Lightweight Shared Multi-Scale Group Conv Detection Head), with its MSGConv-based multi-scale feature extraction capabilities, captures the fine details of small barcodes, allowing for effective detection even in low-resolution or distant scenarios. The LSMD detection head enhances the detection accuracy and efficiency by leveraging shared convolution, which reduces computational redundancy while extracting multi-scale barcode features. This is particularly beneficial when barcodes are confused with similar background elements like lines or patterns, as the LSMD detection head can better distinguish barcodes from irrelevant background objects, reducing false detections. The size, shape, and orientation of barcodes can vary significantly across different scenarios, especially in applications like logistics and retail where multiple barcodes need to be scanned quickly. The MSGConv design in the LSMD detection head ensures the flexible and accurate detection of barcodes at varying scales, ensuring both large and small barcodes are accurately detected.

## 3. Experiment

### 3.1. Dataset Construction and Experimental Environment

The images used in this study were partially collected from the internet and partially taken offline, resulting in a total of 2651 images containing barcodes. The collected images were processed to simulate conditions such as dirt, damage, occlusion, and blur. Among these images, 1758 were allocated to the training set, 393 to the validation set, and 500 to the test set. Manual annotations were performed using the LabelImg tool, and the complex background barcode dataset was made publicly available at https://github.com/yewumingyue0/dataset-code (accessed on 23 November 2024).

The experiments were conducted on a Windows 11 operating system with an Intel^®^ Core™ i7-12650H CPU and an NVIDIA GeForce RTX 4060 GPU. The experimental environment included Pytorch 2.2.2, Python 3.11.8, and CUDA 12.1. Key parameters for model training are shown in Table 1.

### 3.2. Evaluation Indicators

For lightweight barcode detection networks, a comprehensive set of evaluation metrics is necessary to assess both the accuracy and complexity of the model. This study uses the following metrics: Mean Average Precision at 50% IoU (MAP50), Mean Average Precision at IoU from 50% to 95% (MAP50:95), Precision (*P*), Recall (*R*), Model Parameters (Params), Computational Complexity (GFLOPs), Frames Per Second (FPS), and Model Size. The formulas for Precision (*P*) and Recall (*R*) are as follows:

(12) $$\left\{\begin{array}{l} P=\frac{TP}{(TP+FP)}\\ R=\frac{TP}{(TP+FN)} \end{array}\right.$$

where *TP* denotes true positive predictions, *FP* denotes false positive predictions, and *FN* denotes false negative predictions. The formula for Mean Average Precision (MAP) is as follows:

(13) $$MAP=\frac{1}{k}\sum_{i=1}^{k}\left(\int_{0}^{1}P_{i}(R)dr\right)$$

where *i* denotes the *i*-th class in the dataset and *k* denotes the number of classes in the dataset. MAP50 represents the average precision for all classes at an IOU threshold of 0.5. MAP50:95 represents the average precision calculated across IOU thresholds from 0.5 to 0.95, with a step size of 0.05, and is the mean of the *MAP* values obtained at each IOU threshold.

### 3.3. Experimental Analysis

#### 3.3.1. Analysis of Network Improvement Effectiveness

1. Effectiveness Analysis of the MSG-C2f Module

This paper designs the MSG-C2f module based on MSGConv and replaces the original C2f module in the P5 layer of the backbone network. To validate the effectiveness of the MSG-C2f module, comparative experiments are conducted using different feature extraction modules.

From Table 2, it can be observed that compared to the baseline network, the MSG-C2f module improves both the Mean Average Precision MAP50 and MAP50:95 while reducing the number of parameters. Additionally, it demonstrates significant advantages over other modules in terms of MAP50 and recall rate R. Notably, C2f-DCNv3 also achieves commendable results in barcode detection, with its own strengths and weaknesses compared to the proposed MSG-C2f. However, MSG-C2f is specifically designed to address the issue of significant scale variation in barcodes, with the MSGConv design enabling it to more effectively capture features across different scales, which is crucial for barcode recognition. By integrating the MSG-C2f module into YOLOv8, the model’s ability to detect one-dimensional barcodes in complex environments is significantly enhanced.

2. Effectiveness Analysis of the GRCE Module

By applying the GRCE module to replace the original C2f module in the neck structure, the network model not only becomes more flexible but also improves feature extraction capabilities. This study compares several commonly used feature extraction modules with the GRCE module through experiments to verify its effectiveness in detecting barcodes under complex background conditions.

As shown in Table 3, the introduction of the GRCE module significantly reduces the model’s parameter count and computational complexity compared to the original network. In addition, both MAP50 and MAP50:95 were improved compared to the baseline network. This indicates that the GRCE module is more effective in capturing and preserving feature information when handling barcodes in complex environments.

3. Effectiveness Analysis of the LSMD Detection Head

To validate the effectiveness of the proposed LSMD detection head, experiments were conducted comparing it with different detection heads.

As shown in Table 4, the proposed LSMD detection head demonstrates a better balance between accuracy and recall compared to other detection heads, and performs exceptionally well in terms of average precision MAP50 and MAP50:95. This validates the effectiveness of the LSMD detection head for detecting barcodes in complex backgrounds.

#### 3.3.2. Ablation Experiment

To validate the rationale of the proposed algorithm, ablation experiments were designed for analysis. The MSG-C2f module is represented by A, the GRCE module by B, and the LSMD detection head by C. The original YOLOv8-n model serves as the baseline, and new modules are added sequentially. The experimental results are shown in Table 5.

From Table 5, it can be observed that the accuracy of the proposed algorithm improves with the addition of each enhancement. After introducing the MSG-C2f module, compared to the baseline network, the average precision (MAP50 and MAP50:95) increased by 1.67% and 0.79%, respectively, with a reduction in both parameter count and computation complexity. Introducing the GRCE module and LSMD detection head sequentially further improves the accuracy and reduces the parameter count. Specifically, the inclusion of the GRCE module significantly reduces parameters and computation complexity, with MAP50 and MAP50:95 increasing by 1.19% and 0.13%, respectively, compared to the baseline network. The enhanced network shows significant advantages in multi-scale feature extraction and complexity adjustment, leading to improved detection capabilities in complex environments.

The MGL-YOLO algorithm considerably reduces the model size and improves the average precision (MAP50 and MAP50:95), achieving excellent performance across various metrics. However, due to increased algorithm complexity, there is a slight reduction in FPS. The experimental results demonstrate that the proposed improvements provide substantial benefits for detecting barcodes in complex environments.

#### 3.3.3. Comparative Experiments

To comprehensively evaluate the reliability of the proposed algorithm and ensure the results are objective and fair, different object detection networks were selected for comparison. The experimental results are shown in Table 6.

From Table 6, it can be observed that the proposed algorithm outperforms others in terms of average precision mean MAP50 and MAP50:95, achieving a desirable balance across various metrics. Compared to the classic two-stage object detection algorithm, Faster R-CNN, it shows a higher detection accuracy. It also has a clear advantage over the single-stage object detection algorithm SSD. Additionally, compared to YOLOv8-s, it has fewer parameters and higher detection precision. The experimental results indicate that the proposed improvements still hold advantages over other object detection networks.

#### 3.3.4. Visualized Analysis

**Figure 15:**
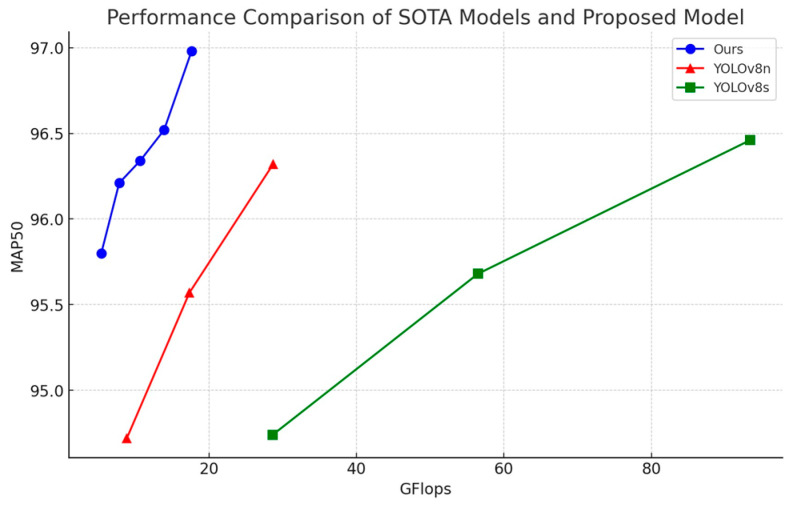
Performance comparison between SOTA models and the proposed model at different input sizes.

This study conducts experiments and tests on YOLOv8n, YOLOv8s, and the proposed model using different input sizes. For the proposed model, input sizes of 640, 768, 896, 1024, and 1152 were used, while YOLOv8n and YOLOv8s were tested with input sizes of 640, 896, and 1152. To ensure the smooth progress of the experiment, a batch size of 8 was selected for this part of the study, differing from previous experimental settings. The results are shown in Figure 15, where the *x*-axis represents GFLOPs and the *y*-axis represents MAP50. As illustrated in the figure, the proposed model demonstrates advantages in both MAP50 and computational efficiency.

**Figure 16:**
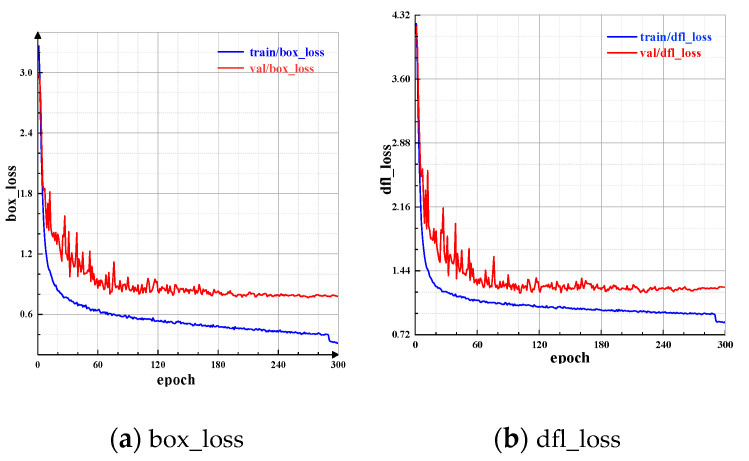
Bounding box loss and feature point loss variation. The box_loss measures the accuracy of predicted bounding boxes compared to ground truth, while dfl_loss assesses the accuracy of predicted feature points. The decrease in both losses over time indicates the model’s improving capability to accurately locate and classify barcodes during training.

When training the MGL-YOLOv8 network, several key metrics reflect the model’s training performance. Box_loss measures the difference between the predicted bounding boxes and the ground truth boxes. By minimizing the box_loss, the model can learn more accurate bounding box locations. Dfl_loss calculates the difference between the predicted feature points and the ground truth feature points. Minimizing dfl_loss helps the model learn more accurate object orientation and angle information. The changes in box_loss and dfl_loss during training and validation are shown in Figure 16.

**Figure 17:**
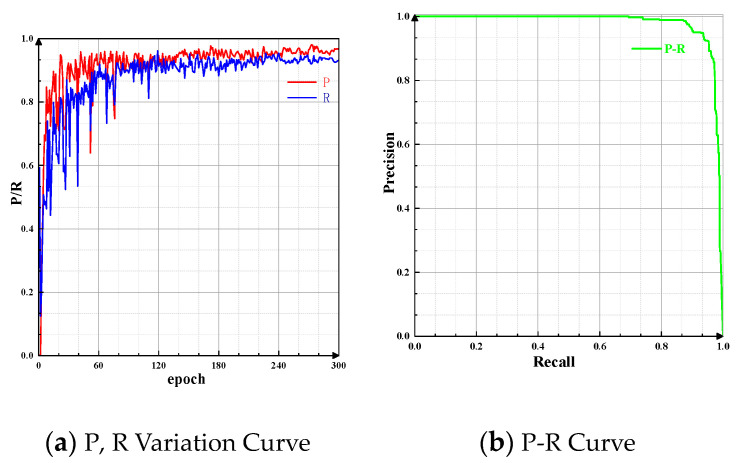
P and R variation and relationship diagram. The P-R curve visually represents the trade-off between precision and recall, with the area under the curve indicating the model’s overall performance. A higher area signifies better detection capability, crucial for assessing the effectiveness of the barcode detection algorithm.

The precision (P) reflects the accuracy of the detection, while the recall (R) indicates the model’s ability to identify instances of objects. The relationship between precision and recall can be represented using the P-R curve, where the area enclosed by the P-R curve and the coordinate axes is defined as the average precision (AP). The variations in P and R, as well as the P-R curve, are shown in Figure 17.

**Figure 18:**
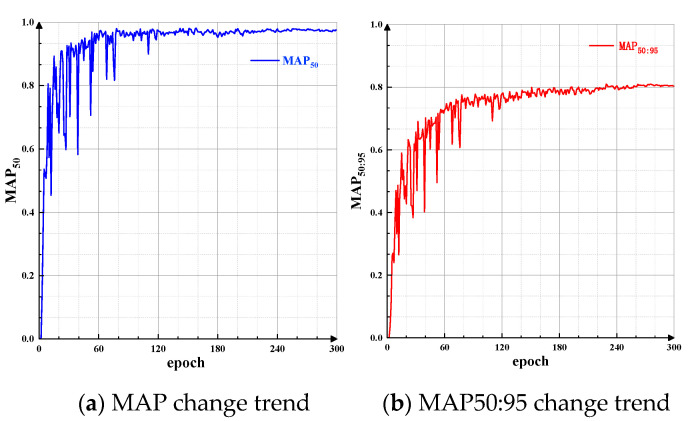
MAP50 and MAP50:95 variation. These metrics are critical for evaluating the model’s detection performance at different Intersection over Union (IoU) thresholds. An increase in these values suggests that the model is becoming more accurate in detecting barcodes, especially under challenging conditions.

The Mean Average Precision (MAP50 and MAP50:95) is used to evaluate the accuracy of object detection by the model. The changes in MAP50 and MAP50:95 are shown in Figure 18.

**Figure 19:**
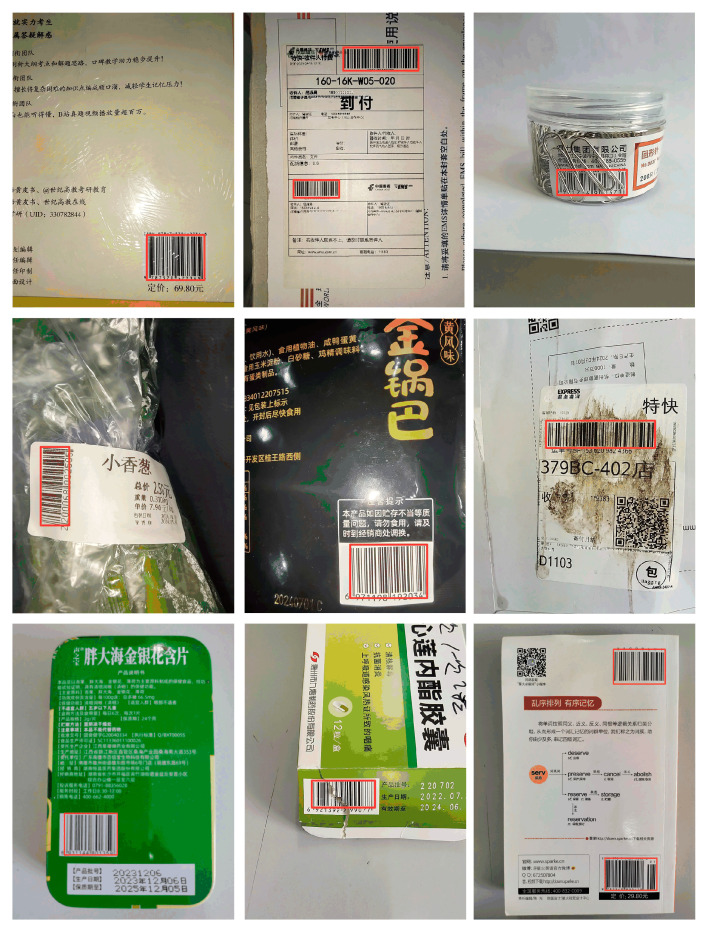
Detection performance in different scenarios. This image showcases the detection performance of the MGL-YOLO algorithm across various scenarios, including logistics and supermarkets. Additionally, it highlights the detection effectiveness under complex conditions such as occlusion, damage, dirt, blurriness, and multiple barcodes. It emphasizes the model’s ability to accurately identify barcodes in diverse environments, demonstrating its robustness and generalization capabilities in practical applications.

This study selected common real-life scenarios such as logistics and supermarkets for detection, and additionally included performance evaluations under conditions such as blurriness, damage, dirt, occlusion, and multiple barcodes. This approach demonstrates the proposed algorithm’s generalization ability across various environments. As shown in Figure 19, the algorithm performs well when detecting objects in different scenarios.

**Figure 20:**
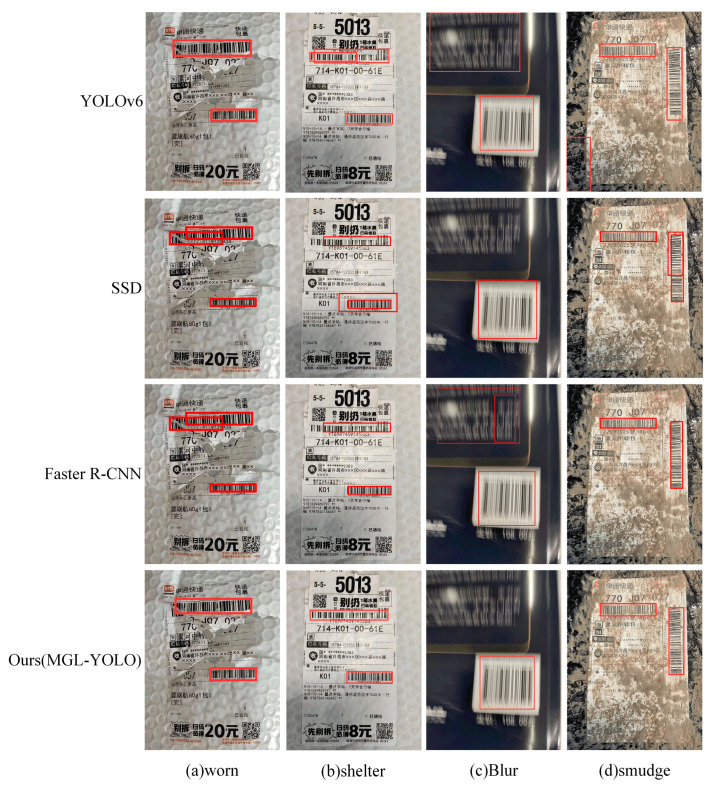
Comparison of detection results in different complex scenarios. This image compares the barcode detection results under various conditions, including damage, occlusion, and blur, across different models such as MGL-YOLO, YOLOv6, SSD, and Faster R-CNN. It highlights the strengths of MGL-YOLO for accurately detecting barcodes in challenging environments, where other models may struggle with repeated detections or misclassifications.

To further evaluate the detection performance of MGL-YOLO, comparisons are made with YOLOv6, SSD, and Faster R-CNN under conditions of dirt, damage, occlusion, and blur. The detection results under different images are illustrated in Figure 20.

From Figure 19, it can be observed that MGL-YOLO is capable of detecting barcodes in all scenarios with varying complexity. In Figure 20a, Faster R-CNN and SSD may detect the same damaged barcode multiple times. As shown in Figure 20b, YOLOv6 and SSD exhibit repeated detections in the case of occlusion. In Figure 20c, Faster R-CNN and YOLOv6 mistakenly identify text as barcodes in blurry scenes. Figure 20d illustrates YOLOv6 incorrectly identifying dirty areas as barcodes and SSD exhibiting repeated detections. The comparison results indicate that the proposed MGL-YOLO algorithm exhibits strong barcode detection capabilities in various environments.

MGL-YOLO outperforms other models in complex scenarios due to its innovative multi-scale feature extraction and optimization mechanisms. The MSG-C2f module’s multi-scale group convolution allows the model to better capture barcode features across different sizes and backgrounds, excelling particularly in challenging conditions such as occlusion, damage, and blur. The GRCE module’s long-range attention mechanism enhances barcode recognition in complex backgrounds, significantly reducing false positives and missed detections. Additionally, the LSMD detection head, with its shared convolution and RepConv structure, improves inference speed and feature extraction efficiency, delivering exceptional performance in both real-time processing and accuracy. As a result, MGL-YOLO offers a distinct advantage over other models in handling complex barcode detection tasks.

## 4. Conclusions

For the problem of large and less accurate network models for barcode detection in complex backgrounds, which cannot be applied to edge detection devices, this paper proposes a lightweight and high-precision MGL-YOLO target detection algorithm based on YOLOv8. Firstly, a multi-scale grouped convolution MSGConv is introduced and applied to the C2f module to design the MSG-C2f module, enhancing the backbone network’s ability to extract multi-scale features. Secondly, the GRCE feature extraction module is proposed to increase model flexibility and significantly reduce the number of parameters. Finally, a lightweight detection head LSMD that utilizes shared multi-scale grouped convolutions is introduced, further reducing the model’s parameters and improving the detection accuracy. Experimental results demonstrate that the proposed lightweight MGL-YOLO network performs excellently at barcode detection in complex environments. The designed modules have a smaller computational and parameter footprint, which is beneficial for applications on memory-constrained edge devices.

Although the proposed algorithm has many advantages, achieving higher accuracy and lightweight objectives has increased the model’s structural complexity, resulting in a reduction in detection speed. Further research is needed to achieve a better balance in the future.

## Figures and Tables

**Table 1:** Training Parameter Settings. These settings are crucial for ensuring the model converges effectively during training and achieving optimal performance on the barcode detection tasks.

Parameter Name	Parameter Setting Value
Lr0	0.01
epochs	300
Batch_size	64
optimizer	AdamW
weight_decay	0.0005
Mosaic	1.0

**Table 2:** Effectiveness Analysis of the MSG-C2f Module. Metrics include MAP50, MAP50:95, precision, recall, parameter count, GFLOPs, model size, and frames per second (FPS). The results indicate that the MSG-C2f module enhances detection accuracy while maintaining a lower parameter count, demonstrating its effectiveness in complex barcode detection environments. The bold part represents the metric with the best performance.

Model	MAP50%	MAP50:95%	P%	R%	Params	GFLOPs	Model Size	FPS
Base (C2f)	95.17	78.38	**97.23**	89.57	3.01 M	8.2	5.98 M	**265.9**
C2f-Faster [22]	96.76	79.22	95.49	91.35	**2.79 M**	**8.0**	**5.56 M**	265.1
C2f-DEConv [23]	94.36	77.46	96.09	87.53	3.40 M	8.1	6.74 M	217.0
C2f-DCNv3 [24]	96.44	**79.37**	95.24	91.58	2.90 M	8.1	5.77 M	243.1
C2f-DySnakeConv [25]	96.14	78.69	95.96	90.59	3.27 M	8.3	6.49 M	161.0
Ours (MSG-C2f)	**96.84**	79.17	93.35	**92.80**	2.97 M	8.2	5.91 M	256.8

**Table 3:** Effectiveness analysis of the GRCE module. The results highlight the GRCE module’s ability to improve feature extraction capabilities and reduce computational complexity, making it suitable for detecting barcodes in challenging backgrounds. The bold part represents the metric with the best performance.

Model	MAP50%	MAP50:95%	P%	R%	Params	GFLOPs	Model Size	FPS
Base (C2f)	95.17	78.38	**97.23**	89.57	3.01 M	8.2	5.98 M	265.9
C2f-Star [26]	96.14	79.48	95.48	90.59	2.71 M	7.3	5.40 M	268.4
BottleneckCSP [27]	95.49	78.09	96.35	89.06	2.99 M	8.1	34.7 M	240.8
C2f-AKConv [28]	95.36	78.47	95.58	89.57	2.87 M	8.2	5.45 M	70.8
RepBlock [29]	96.12	**79.98**	96.98	90.02	3.35 M	8.4	6.67 M	261.1
C2f-OREPA [30]	96.36	78.42	95.27	90.17	4.13 M	**7.3**	8.24 M	266.8
Ours (GRCE)	**96.37**	78.53	95.69	**90.84**	**2.63 M**	7.4	**5.26 M**	**274.5**

**Table 4:** Effectiveness analysis of the LSMD module. The results demonstrate that the LSMD head balances accuracy and efficiency effectively, making it a strong candidate for real-time barcode detection applications. The bold part represents the metric with the best performance.

Model	MAP50%	MAP50:95%	P%	R%	Params	GFLOPs	Model Size	FPS
Base	95.17	78.38	97.23	89.57	3.01 M	8.2	5.98 M	265.9
Detect_NMSFree [31]	94.92	75.51	**97.76**	88.72	3.76 M	11.3	7.51 M	255.8
DetectAux [32]	96.07	75.92	94.59	88.28	3.76 M	11.2	7.43 M	**270.6**
RTDETRDecoder [33]	70.84	52.86	80.03	64.12	9.48 M	16.8	18.3 M	86.9
DyHead [34]	**96.73**	78.70	94.72	91.15	3.63 M	10.5	6.90 M	130.5
Ours (LSMD)	**96.73**	**79.56**	95.48	**91.29**	**2.34 M**	**6.3**	**4.70 M**	193.6

**Table 5:** Ablation Experiment. The incremental improvements shown in the results validate the importance of each module in enhancing detection capabilities. The bold part represents the metric with the best performance.

A	B	C	MAP50%	MAP50:95%	P%	R%	Params	GFLOPs	Model Size	FPS
			95.17	78.38	**97.23**	89.57	3.01M	8.2	5.98M	265.9
√			96.84	79.17	93.35	92.80	2.97M	8.2	5.91M	256.8
	√		96.37	78.53	95.69	90.84	2.63M	7.4	5.26M	**274.5**
		√	96.73	79.56	95.48	91.29	2.34M	6.3	4.70M	193.6
√	√		96.62	78.7	95.54	92.65	2.59M	7.4	5.19M	265.6
√		√	97.38	78.56	95.77	92.07	2.30M	6.2	4.63M	189.8
	√	√	97.42	78.36	94.77	92.22	1.97M	5.5	3.99M	200.0
√	√	√	**97.74**	**80.69**	95.10	**93.74**	**1.92M**	**5.4**	**3.92M**	190.0

**Table 6:** Comparative experiments with other networks. The results highlight MGL-YOLO’s superior accuracy and efficiency, establishing its competitiveness in the field of barcode detection. The bold part represents the metric with the best performance.

Model	MAP50%	MAP50:95%	P%	R%	Params	GFLOPs	Model Size	FPS
Faster R-CNN [35]	97.7	74.0	79.93	**97.54**	136.8 M	369.8	521 M	25.9
YOLOv6-n [36]	96.1	76.9	96.1	93.4	4.63 M	11.34	9.97 M	208.3
YOLOv7-tiny [32]	89.9	64.4	94.9	81.1	6.01 M	13.2	11.7 M	222.2
YOLOv8-n [37]	95.17	78.38	**97.23**	89.57	3.01 M	8.2	5.98 M	**265.9**
YOLOv8-s [37]	95.66	78.53	93.89	89.31	11.13 M	28.6	21.4 M	192.0
SSD [38]	94.55	67.1	96.12	90.91	24.1 M	61.2	90.6 M	121.3
ASF-YOLO [39]	96.0	78.7	93.6	91.4	47.1 M	116.6	90.3 M	57.4
GOLD-YOLO [40]	96.1	74.7	96.8	91.3	5.6 M	12.1	177 M	83.2
RCS-YOLO [41]	92.2	74.4	95.8	92.1	50.59 M	105.2	96.8 M	85.9
Mamba-YOLO [42]	95.7	70.7	95.0	87.0	5.99 M	14.1	11.7 M	82.9
YOLO-QR [43]	96.4	78.2	97.1	90.1	30.99 M	89.2	59.4 M	69.5
MobileNetv4 [44]	96.03	77.61	95.8	87.13	2.94 M	9.87	11.2 M	191.4
GhostNetv2 [45]	96.78	80.07	94.47	93.13	2.31 M	6.9	4.7 M	219.5
Ours (MGL-YOLO)	**97.74**	**80.69**	95.10	93.74	**1.92 M**	**5.4**	**3.92 M**	190.0

## Data Availability

Dataset: https://github.com/yewumingyue0/dataset-code.
